# Variations in Assessment Methods of Heterogeneous Exercise Capacity Response After Cardiac Rehabilitation

**DOI:** 10.3390/jcm15218662

**Published:** 2026-11-06

**Authors:** Małgorzata Kurpaska, Katarzyna Piotrowicz, Marzena Kubiak, Katarzyna Alisz, Paweł Krzesiński

**Affiliations:** Department of Cardiology and Internal Diseases, Military Institute of Medicine, National Research Institute, Szaserow Street 128, 04-141 Warsaw, Poland

**Keywords:** cardiac rehabilitation, exercise improvement, oxygen uptake, six-minute walk test, metabolic equivalent

## Abstract

**Background:** Cardiac rehabilitation (CR) improves the prognosis of patients with cardiac diseases by, among other measures, improving exercise capacity (EC) as assessed according to different parameters. **Objective:** This study aimed to evaluate the effects of phase II of CR on the different measurements of EC and to assess the distribution of changes in these measurements among patients who underwent the intervention. **Methods:** From a single-center retrospective registry, records of 295 patients who underwent the six-minute walk test (6MWT) and cardiopulmonary exercise testing twice during CR were evaluated. The distance of the 6MWT, peak oxygen uptake (VO_2_) expressed in absolute values and as a percentage of predicted values, and workload on a cycle ergometer were assessed before and after CR and then analyzed. **Results:** Among the 295 patients (228 men, mean age 62 years [range: 55–69 years]), CR resulted in improvements in the median value of peak VO_2_ (approximately 1 mL/kg/min, 3% of the predicted value), workload (by approximately 5 watt), and 6MWT distance (by approximately 24 m, 5% of the predicted value). A total of 53.6% of the patients achieved an improvement in peak VO_2_ of ≥1 mL/kg/min and 44.1% of the patients achieved an improvement in 6MWT distance of >30 m. Simultaneous improvement in both peak VO_2_ and 6MWT distance was observed in 23% of the participants, whereas 26% did not achieve clinically meaningful improvement in either parameter. **Conclusions:** CR improved exercise capacity, although the proportion of patients who achieved clinically meaningful improvement differed substantially across the exercise capacity measurements.

## 1. Introduction

Cardiac rehabilitation (CR) is expected to improve exercise capacity (EC) [1,2], quality of life [1,3], and, most importantly, prognosis in patients with cardiovascular diseases [1,4,5]. However, participation in CR alone and maintaining at least moderate physical activity for 30 min per day—a therapeutic goal [5]—appear insufficient for assessing the CR effect. Following the completion of the planned training sessions during phase II of CR, reassessment of EC is recommended after two to three months, whereas during phase III CR, annual assessment is advised [1,6].

Due to differences in the capabilities of medical centers and the organization of CR programs, EC may be evaluated after different training durations (from six weeks to three months or even one year) and using different parameters [1,2], including changes in peak oxygen uptake (peak VO_2_), metabolic equivalents of task (METs), or the distance covered during the six-minute walk test (6MWT). Among these parameters, improvements in peak VO_2_ [1,7,8] and METs [3,9] have been most consistently associated with improved prognosis. In contrast, changes in 6MWT distance have been shown to correlate with improvements in health-related quality of life (HRQoL) [2] and patients’ subjective perceptions of improved EC [10].

Most related studies have demonstrated significant improvements in EC measurements during CR [1]. The 2026 ESC guidelines [1] indicate the need to achieve minimum therapeutic targets of a 1 mL/min/kg increase in peak VO_2_ and a 30–45 m increase in 6MWT distance [1]. However, data regarding the proportion of patients who achieve these therapeutic targets and changes in EC measurements remain limited. Determining the proportion of patients who respond to exercise training may facilitate individualized treatment, identify patient groups that require modification of rehabilitation strategies, and help determine the factors that influence therapeutic outcomes. Assessment of the training effects on EC is essential when CR is considered a continuous care model (phases II and III) rather than a one-time intervention [1,11,12]. Thus, the aim of this study was to evaluate the effects of phase II of CR on different measurements of EC and to assess the distribution of changes in these measurements among the patients who underwent the intervention.

## 2. Methods

A single-center retrospective study was conducted of patients who underwent CR between 2022 and 2024. The exclusion criteria consisted of a history of cardiac surgery and the absence of two cardiopulmonary exercise tests (CPETs) performed during CR. The medical records that were collected at the time of CR enrollment were analyzed, including demographic characteristics, comorbidities, reported symptoms and physical findings, and pharmacological treatments. Echocardiographic data were obtained from examinations performed during an acute coronary event or qualification for coronary angiography/coronary angioplasty.

Before CR, laboratory assessments included hemoglobin, creatinine, and glucose concentrations. Hemoglobin levels < 12 g/dL in women and <13 g/dL in men were considered diagnostic of anemia [13]. The glomerular filtration rate (GFR) was calculated using the Cockcroft–Gault equation. Body weight was measured, and body mass index (BMI) was calculated. Obesity was defined as BMI ≥ 30 kg/m^2^.

CPET was performed using a stationary cycle ergometer (Geratherm Respiratory GmbH; Bitz, Germany) with breath-by-breath gas exchange analysis (Biophysik GmbH; Leipzig, Germany). Each patient underwent two CPETs using the same protocol: one at CR enrollment and the second after completion of CR. An individualized ramp protocol was applied, with workload increasing linearly from 0 watts to the predicted maximal workload calculated according to the Wasserman equation over approximately ten minutes [14]. Exercise was terminated at the patient’s request. Data were averaged over 30 s intervals.

Peak VO_2_ was measured, and the percentage of predicted peak VO_2_ was calculated using the Hansen reference equations [14]. An increase in peak VO_2_ of ≥1 mL/kg/min was considered the minimal clinically important improvement after six–seven weeks of CR [7,8]. Reduced EC was defined as peak VO_2_ < 85% of the predicted value [15].

The minute ventilation–carbon dioxide production relationship (VE/VCO_2_ slope) was calculated between the first ventilatory threshold and the respiratory compensation point (second ventilatory threshold) [14]. The workload achieved on the ergometer during CPET was assessed and converted into METs using the American College of Sports Medicine metabolic equations [16]; a value of ≥1 was considered the minimum clinically significant change after six–seven weeks [1,3,9,17].

We also assessed the distance covered in the 6MWT and the percentage of the predicted distance achieved in the 6MWT [18]. A difference of ≥30 m between the distance achieved in the second 6MWT before CR (repeated within three days) and the distance achieved after the completion of rehabilitation was considered clinically significant [19,20].

The analysis included patients who participated either in an inpatient CR program (10–14 supervised exercise sessions) or in a hybrid CR program (29–34 exercise sessions supported by telemonitoring). Exercise training was performed with intensity individually prescribed according to each patient’s clinical status with low, moderate, or high intensity based on the Karvonen formula or ventilatory threshold [6]. Inpatient rehabilitation consisted of continuous exercise on a cycle ergometer, whereas hybrid rehabilitation was based on interval training on a cycle ergometer or walking.

The study was conducted in accordance with the Declaration of Helsinki and approved by the Institutional Review Board (IRB) of the local Institute (Resolution 32/24 from 19 June 2024). The need for informed consent was waived by the IRB due to the retrospective nature of the study.

The statistical analyses were performed using Statistica software, version 12.0 (TIBCO Software Inc., Palo Alto, CA, USA). The distribution of continuous variables was evaluated with the Shapiro–Wilk test. Continuous variables with a normal distribution were expressed as means ± SD, and those with a non-normal distribution were expressed as medians and interquartile ranges (IQR: Q1–Q3). Changes in peak VO_2_, peak workload, METs, and 6MWT distance between the baseline and follow-up assessments were calculated. A non-parametric Wilcoxon test was used to assess the statistical significance of the changes in the assessed parameters during CR in the entire study population. Associations between changes in peak VO_2_ and 6MWT distance were assessed using Spearman’s rank correlation coefficient. Agreement between responder classifications based on clinically meaningful improvements in peak VO_2_ (≥1 mL/kg/min) and 6MWT distance (≥30 m) was evaluated using Cohen’s κ coefficient with asymptotic 95% confidence intervals. A *p*-value < 0.05 (two-tailed) was considered statistically significant.

## 3. Results

Of the 518 patients referred for CR, 52 patients with a history of cardiac surgery and 171 patients who did not undergo two CPETs were excluded. Of the latter, 43 patients did not undergo baseline CPET at enrollment, and 128 patients did not complete the follow-up assessment. Consequently, 295 patients were included in the final analysis for this study. The median age of the patients was 62 (55–69) years, and most of the participants were men (77%).

Almost all of the patients (98%) who underwent CR had a history of coronary artery disease (CAD) treated with coronary angioplasty, and 70% had experienced acute coronary syndrome (Table S1). The most common comorbidities were risk factors for CAD, including hypertension (70%) and dyslipidemia (73%), while type 2 diabetes mellitus was less prevalent (26%). Heart failure (HF) was diagnosed in 37% of the patients (Table S1).

Nearly half of the patients (49%) reported an unspecified reduction in exercise tolerance before CR, including exertional dyspnea in 17% and chest pain in 9%. Furthermore, 98% of patients were taking statins, 92% were treated with ACEIs/ARBs or ARNIs, and 87% were taking beta-blockers (Table S2).

At least mildly reduced EC was observed in 62% of the patients enrolled for CR; the median peak VO_2_ was 81% of the predicted value. Only 17% of the subjects exhibited ventilatory inefficiency corresponding to class II (VE/VCO_2_ slope > 36). Nevertheless, the median 6MWT distance was 100% of the predicted value.

Following the completion of phase II of CR, the study group showed significant improvement in EC, including peak VO_2_ (19 versus [16,17,18,19,20,21,22] 20 mL/kg/min [16,17,18,19,20,21,22,23,24]; *p* < 0.0001), predicted peak VO_2_ (81% [70–91%] versus 84% [72–95%]; *p* < 0.0001), 6MWT distance (569 [507–634] m versus 593 [542–659] m; *p* < 0.0001), and workload (125 [94–159] versus 130 [100–166] Watts; *p* < 0.0001) (Table 1 and Table S3, Figure S1). The prevalence of reduced EC (<85% of predicted peak VO_2_) decreased significantly (62% versus 51%; *p* = 0.007). Compared to the 6MWT distance achieved before CR, 21% (57) more patients achieved the predicted 6MWT distance (Table 1). No significant changes in VE/VCO_2_ slope or RER values were observed during CR.

**Figure 1:**
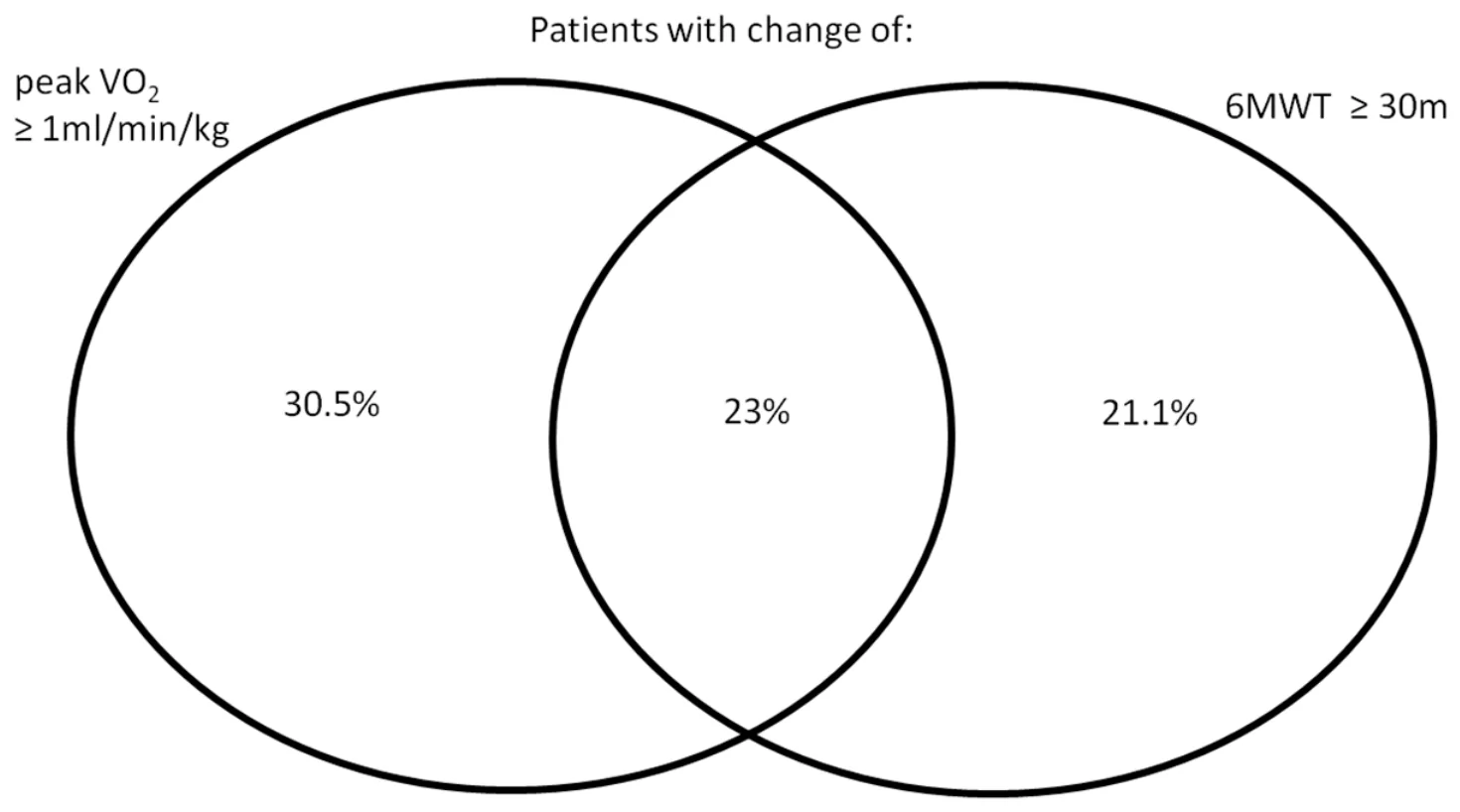
Number of patients who achieved the minimally important clinical improvements according to the parameters (based on clinically meaningful improvements in peak VO_2_ (≥1 mL/kg/min) and 6MWT distance (≥30 m)).

**Figure 2:**
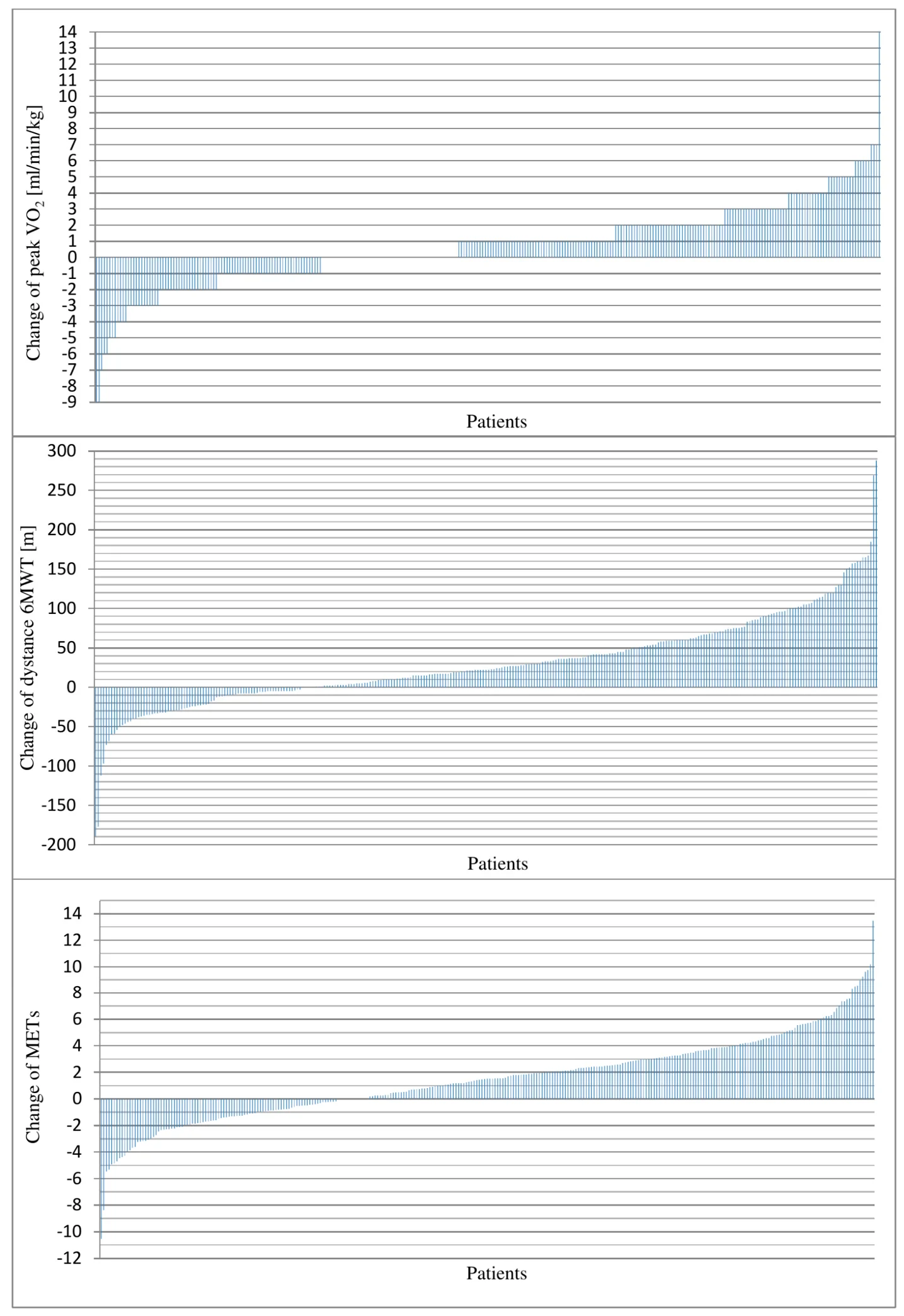
Improvements in the patients’ exercise performance according to the parameters.

Among the 295 patients in the study group, 53.5% (158) achieved an improvement in EC—expressed as peak VO_2_—of ≥1 mL/kg/min. An improvement in METs of ≥1 was observed in 56.3% (166) of the patients, and 44.1% (130) achieved an improvement in the 6MWT distance of >30 m (Figure 1). Importantly, the pattern of improvement across the assessed EC parameters was highly heterogeneous (Figure 2). Clinically meaningful improvement in peak VO_2_ alone was observed in 30.5% of the patients, in the 6MWT distance alone in 21.1% of the patients, and in both of these measurements in 23% (68) of the patients (Figure 1). As many as 25.5% (75) did not achieve clinically meaningful improvement in either peak VO_2_ or 6MWT distance. No significant correlation was observed between changes in peak VO_2_ and 6MWT distance (Spearman’s ρ = 0.037, *p* = 0.542). Agreement between responder classifications was negligible (Cohen’s κ = −0.027, 95%CI: −0.143 to 0.088), with concordant classifications in 48.2% of patients.

## 4. Discussion

CR improved EC at the population level in the overall statistics. However, the response to rehabilitation differed substantially according to the parameter used to assess exercise performance. While the median values of peak VO_2_, workload, and 6MWT distance all improved significantly, clinically meaningful improvement was achieved by different proportions of patients depending on the assessment parameter. Notably, less than one quarter of the participants demonstrated simultaneous improvement in both peak VO_2_ and 6MWT distance, whereas one quarter did not achieve clinically meaningful improvement in either parameter. These findings suggest that different measures of EC identify distinct patterns of response to CR, which may reflect both differences in the aspects of exercise performance assessed and the measurement characteristics of each test. The lack of a significant correlation between changes in peak VO_2_ and 6MWT distance, together with negligible agreement between responder classifications, further supports the complementary nature of these assessment methods. However, these discrepancies may reflect differences in test characteristics, sensitivity to change, baseline functional capacity, and thresholds for clinically meaningful improvement, rather than necessarily indicating distinct physiological adaptations.

Following CR, significant improvements in EC were observed, consistent with previous reports that demonstrated the overall effectiveness of CR [1]. However, our findings suggest that different exercise parameters capture complementary domains of recovery rather than a uniform training response.

Peak VO_2_ is one of the strongest prognostic markers in cardiovascular disease. An increase of 1 mL/kg/min has been associated with an approximately 10% reduction in cardiovascular mortality [7,8], while even smaller improvements have been linked to lower risks of death and hospitalization [21]. However, these prognostic associations were established in previous studies and should not be directly extrapolated to our findings, as long-term clinical outcomes were not assessed. In our study group, 53.6% of patients achieved an increase in peak VO_2_ of ≥1 mL/kg/min, a lower proportion than previously reported in HF with reduced ejection fraction after acute coronary syndrome [22], coronary artery disease [23], and predominantly post-coronary intervention populations [24]. These differences are likely explained by variations in patient characteristics, rehabilitation duration, exercise intensity, and definitions of clinically meaningful improvement [21,25]. Our study was conducted with an unselected contemporary CR population with predominantly hybrid rehabilitation, which reflected our routine clinical practice. Previous studies showed that older age, higher baseline EC, peripheral artery disease, a low level of physical activity, fewer completed training sessions, diabetes, female sex, and low training intensity were all associated with a lower probability of improving peak VO_2_ [22,23,24].

The 6MWT reflects functional performance and is widely used during CR because of its simplicity and clinical relevance [1]. Although the minimal clinically important improvement has been estimated at approximately 25–30 m [19,20,25,26], our patients achieved a median improvement of 24 m, smaller than the 59–75 m reported previously [2,20,27,28]. A likely explanation is the high baseline functional status of our study group, with a median walking distance that already reached 100% of the predicted value and left limited room for further improvement. This ceiling effect may also explain why fewer patients achieved clinically meaningful improvement in the 6MWT than in peak VO_2_, despite the moderate correlation between the two measures [28]. Interestingly, one fifth of patients improved only in the 6MWT despite no significant change in peak VO_2_, which suggested that walking performance may capture functional adaptations relevant to daily activities and HRQoL that are not fully reflected by CPET [2,10]. These discrepancies may reflect differences in the physiological domains assessed and the responsiveness of each test rather than necessarily indicating distinct physiological adaptations. While peak VO_2_ reflects integrated cardiovascular and peripheral responses to maximal exercise, the 6MWT assesses self-paced functional walking performance, which is also influenced by pacing, motivation, and test familiarity [22,29]. Importantly, the correlation between 6MWT distance and peak VO_2_ is only moderate, and changes in walking distance may not reliably reflect changes in directly measured peak VO_2_ [28,30]. Differences in measurement sensitivity, particularly at higher baseline functional capacity, and the use of different thresholds for clinically meaningful improvement may therefore contribute to the observed discordance in responder classification.

Because direct measurement of peak VO_2_ is not universally available in routine clinical practice, we also analyzed estimated EC expressed as METs as a more common measure for evaluating functional improvement during CR. In the absence of direct measurement of peak VO_2_, numerous guidelines suggest the possibility of estimating performance using METs [1,12]. In our study group, METs showed the highest proportion of responders (56.3%), closely matching the results obtained for peak VO_2_. This similarity was expected because both parameters are derived from the same exercise test. Although estimated MET values obtained during cycle ergometry may underestimate true metabolic demand compared with treadmill testing [31], they remain clinically useful for repeated within-patient assessment. Previous studies consistently demonstrated that each 1-MET improvement is associated with better prognosis [3,10,17,32], whereas greater improvement is generally observed in patients with lower baseline EC and preserved left ventricular function [3,33].

In contrast, ventilatory efficiency remained unchanged. Although improvements in VE/VCO_2_ slope have been described after exercise training [34,35,36], no significant change was observed in our study, which was consistent with other reports [37]. This may reflect the relatively short duration and moderate intensity of rehabilitation [38], but it also suggests that improvements in functional performance may occur earlier than changes in ventilatory efficiency, which is more strongly influenced by persistent cardiopulmonary and hemodynamic abnormalities [39]. Moreover, in the studies in which no reduction in the VE/VCO_2_ slope was observed [37], the lack of change was attributed to a complex pathomechanism—specifically, that pulmonary limitations were likely independent of hemodynamic or neurohormonal modulation.

Overall, our findings support the concept that response to CR is heterogeneous and depends on the parameter used to assess EC [28,40]. Rather than simply identifying responders and non-responders, different exercise tests appear to identify different response profiles. Future studies should investigate whether these distinct response phenotypes, including different combinations of improvement across CPET- and walking-based measures, are associated with long-term clinical outcomes and may improve risk stratification after CR.

## 5. Study Limitations

This study has several limitations. First, its retrospective, single-center design limits the generalizability of the findings. Second, although nearly 300 patients were included, the sample size was relatively modest for detailed subgroup analyses. Third, the study population was clinically heterogeneous, including patients with different cardiovascular diagnoses, comorbidity burden, baseline EC, and rehabilitation programs. In particular, inpatient and hybrid CR programs differed in the number of exercise sessions and training modalities. Nevertheless, previous evidence suggests comparable improvements in EC between telerehabilitation and center-based CR. A recent meta-analysis found no significant differences in improvements in peak VO_2_ or 6MWT distance [41], while a randomized trial demonstrated the noninferiority of hybrid CR in improving functional capacity [42]. However, differences in training volume, intensity, and exercise modalities may have influenced individual responses in our cohort. As our study was not designed to compare rehabilitation approaches, their potential impact on the observed outcomes could not be determined. Fourth, EC was assessed using performance-based tests that are influenced not only by physiological adaptation but also by patient motivation, familiarity with the testing procedures, and day-to-day variability, particularly for the 6MWT. Furthermore, patients excluded from the analysis were older and had a greater burden of comorbidities than those included (Table S1), indicating potential selection bias that may limit the generalizability of our findings. Finally, the analysis focused on short-term changes during phase II CR without long-term follow-up to determine whether the observed improvements translated into sustained functional benefits or improved clinical outcomes.

## 6. Conclusions

CR improved EC, although the proportion of patients who achieved clinically meaningful improvement differed substantially across the EC measures. Different assessment methods identified different responder profiles, suggesting that no single parameter fully reflects the effects of rehabilitation. The effectiveness of CR requires proper assessment not only of the average EC measurement values but especially of the number of patients demonstrating clinically significant improvements. Future studies should focus on identifying predictors of parameter-specific responses and determining whether these improvements translate into better long-term clinical outcomes.

## Figures and Tables

**Table 1:** Comparison of exercise performance before and after cardiac rehabilitation.

Parameters	Before Cardiac Rehabilitation	After Cardiac Rehabilitation	*p*
Peak workload (watts)	125 (94–159)	130 (100–166)	<0.0001
RER	1.14 (1.08–1.21)	1.14 (1.08–1.21)	0.412
Peak VO_2_ (ml/kg/min)	19 (16–22)	20 (16–24)	<0.0001
Percentage of predicted value peak VO_2_ (%)	81 (70–91)	84 (72–95)	<0.0001
Patients with peak VO_2_ < 85% predicted value	184 (62%)	150 (51%)	0.007
VE/VCO_2_ slope	30.5 (27.2–34.2)	30.7 (28–33.8)	0.309
Patients with peak VE/VCO_2_ slope > 36	50/294 * (17%)	44/286 * (15%)	0.596
Distance of 6MWT (m)	569 (507–634)	593 (542–659)	<0.0001
Percentage of predicted distance of 6MWT (%)	100 (92–108)	105 (97–114)	<0.0001
Patients with distance of 6MWT > 100% of the predicted value	135/292 * (46%)	192/287 * (67%)	<0.0001

Reporting results as median (Q1–Q3) or *n* (%). * the values were not reported for the entire study population. 6MWT—six-minute walk test, RER—respiratory exchange ratio, VE/VCO_2_—slope of ventilatory insufficiency, VO_2_—oxygen uptake.

## Data Availability

The datasets presented in this article are not readily available because the data is covered by traditional medical confidentiality. Requests to access the datasets should be directed to the Military Institute of Medicine—National Research Institute.

## Supplementary Materials

The following supporting information can be downloaded at: https://www.mdpi.com/article/10.3390/jcm15218662/s1, Table S1. Baseline characteristics of included and excluded patients. Table S2. Basic characteristics of pharmacotherapy in the study group. Table S3. Basic characteristics of the patients’ exercise performance. Figure S1. Improvements in exercise measurements (a) oxygen uptake (peak VO_2_), (b) workload, (c) distance of six-minute walk test in the study group.
