# Supplementary material for: Variations in Assessment Methods of Heterogeneous Exercise Capacity Response After Cardiac Rehabilitation
Source: J Clin Med. 2026 Nov 6;15(21):8662. doi: 10.3390/jcm15218662 (PMC13764196; doi:10.3390/jcm15218662)

Supplementary Tables

Supplementary Table S1. Baseline characteristics of included and excluded patients

	**Included (n = 295)**	**Excluded (n = 223)**	**p-value**
Age (years)	62 (55–69)	69 (60.5–74)	<0.001
Body mass index (kg/m²)	27.3 (25.2–29.7)	27.4 (24.8–30.8)	0.498
Obesity (BMI ≥30 kg/m²)	70 (23.7%)	66 (30.4%)	0.091
Male sex	226 (76.6%)	146 (65.5%)	0.005
**Comorbidities**			
Coronary artery disease	290 (98.3%)	204 (91.5%)	<0.001
ST-segment elevation myocardial infarction	78 (26.4%)	40 (17.9%)	0.022
Non-ST-segment elevation myocardial infarction	137 (46.4%)	102 (45.7%)	0.874
Coronary artery bypass grafting	2 (0.7%)	28 (12.6%)	<0.001
Heart failure	108 (36.6%)	144 (64.6%)	<0.001
Heart failure with LVEF <50%	90 (30.5%)	105 (47.1%)	<0.001
Arterial hypertension	206 (69.8%)	175 (78.5%)	0.027
Dyslipidemia	216 (73.2%)	151 (67.7%)	0.172
Type 2 diabetes mellitus	77 (26.1%)	77 (34.5%)	0.038
Atrial fibrillation	27 (9.2%)	59 (26.5%)	<0.001
Chronic obstructive pulmonary disease	25 (8.5%)	24 (10.8%)	0.378
Peripheral artery disease	15 (5.1%)	17 (7.6%)	0.235
Chronic kidney disease	12 (4.1%)	35 (15.7%)	<0.001
Valvular heart disease	42 (14.2%)	61 (27.4%)	<0.001
Anemia (hemoglobin-defined)	58 (19.9%)	98 (45.8%)	<0.001
**Echocardiography**			

	Included (n = 295)	Excluded (n = 223)	p-value
Left ventricular ejection fraction (%)	55 (45–60)	48 (40–57)	<0.001
Left ventricular diastolic dysfunction	94 (31.9%)	83 (37.2%)	0.203
**Laboratory parameters**			
Hemoglobin (g/dl)	13.8 (12.8–14.6)	12.8 (11.6–13.9)	<0.001
Creatinine (mg/dl)	0.90 (0.80–1.10)	1.00 (0.80–1.20)	0.027
Creatinine clearance (Cockcroft–Gault, ml/min)	91.1 (73.5–108.5)	75.7 (59.6–95.4)	<0.001
Data are presented as median (Q1–Q3) or n (%). Continuous variables: two-sided Mann–Whitney U test; categorical variables: Pearson χ^2 test or two-sided Fisher exact test when counts were sparse.			

Supplementary Table S2. Basic characteristics of pharmacotherapy in the study group

Parameters	Study group (n = 295)
Statin drugs	224 (76%)
Statins with ezetimibe	65 (22%)
ACEI/ARB/ARNI	274 (92%)
Beta-blockers	259 (87%)
Calcium blockers	86 (29%)
Diuretic	115 (39%)
Mineralocorticoid receptor antagonists	64 (22%)
Metformin	63 (21%)
Sodium/glucose cotransporter 2 inhibitors	73 (25%)
Amiodarone	15 (5%)
Reporting results as mean ± SD/median (Q1–Q3) or n (%)<br>ACEI - angiotensin-converting enzyme inhibitor, ARB - angiotensin II receptor blocker, ARNI - angiotensin receptor-neprilysin inhibitor, GFR - glomerular filtration rate, NYHA - New York Heart Association classification.	

Table S3. Basic characteristics of the patients' exercise performance

Parameters	Study group (n = 295)
Distance of the 6MWT (m)	569 (507–624)
Percentage of the predicted distance of the 6MWT	100% (92–108)%
VE/VCO_2 slope	30.5 (27.2–34.2)

Patients with VE/VCO_2 slope ≥ 36	50 (17%)
Peak VO_2 (ml/kg/min)	19 (16–22)
Percentage of the predicted peak of VO_2 (%)	81 (70–91)
Patients with peak VO_2 < 85% predicted value	184 (62%)
Peak workload (watt)	125 (94–159)
RER	1.14 (1.08–1.21)
Lower range of training heart rate according to Karvonen formula	36 (27–44)
Upper range of training heart rate according to Karvonen formula	52 (42–63)
Time exercise per day with telemonitoring (min)	32.25 (32.1–34.5)
Reporting results as median (Q1–Q3) or n (%)<br>6MWT - six-minute walk test, RER - respiratory exchange ratio, VE/VCO_2 - slope ventilatory insufficiency, VO_2 - oxygen uptake.	

Supplementary Figure

Figure S1. Improvements in exercise measurements (a) oxygen uptake (peak VO_2), (b) workload, (c) distance of six-minute walk test in the study group.

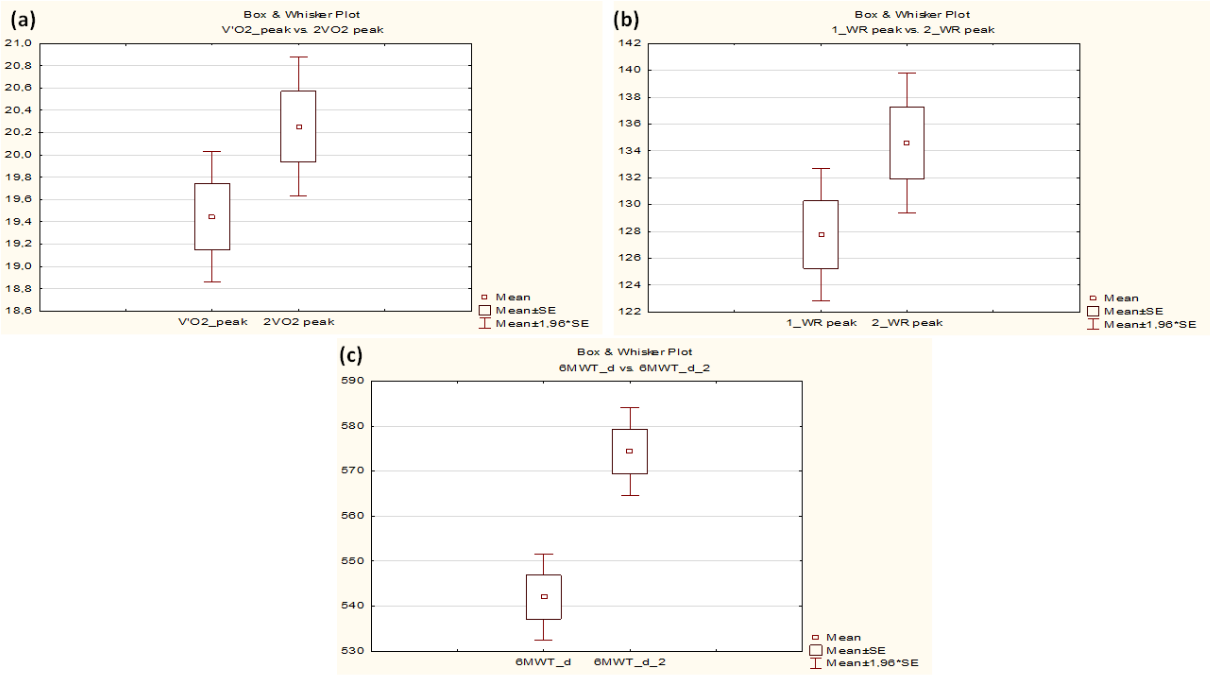

Supplement: Supplementary file 1 [file jcm-15-08662-s001.zip › jcm-4625909-supplementary.pdf]
